# Adenomyosis is an independent risk factor for complications in deep endometriosis laparoscopic surgery

**DOI:** 10.1038/s41598-022-11179-8

**Published:** 2022-04-30

**Authors:** Meritxell Gracia, Cristian de Guirior, Marta Valdés-Bango, Mariona Rius, Cristina Ros, Isabel Matas, Marta Tortajada, María Ángeles Martínez-Zamora, Lara Quintas, Francisco Carmona

**Affiliations:** Endometriosis Unit, ICGON, Hospital Clinic of Barcelona, University of Barcelona, Villarroel 170, 08036 Barcelona, Spain

**Keywords:** Chronic inflammation, Reproductive signs and symptoms

## Abstract

Deep endometriosis (DE) occurs in 15–30% of patients with endometriosis and is associated with concomitant adenomyosis in around 25–49% of cases. There are no data about the effect of the presence of adenomyosis in terms of surgical outcomes and complications. Thus, the aim of the present study was to evaluate the impact of adenomyosis on surgical complications in women with deep endometriosis undergoing laparoscopic surgery. A retrospective cohort study including women referred to the endometriosis unit of a referral teaching hospital. Two expert sonographers preoperatively diagnosed DE and adenomyosis. DE was defined according to the criteria of the International Deep Endometriosis Analysis group. Adenomyosis was considered when 3 or more ultrasound criteria of the Morphological Uterus Sonographic Assessment group were present. Demographical variables, current medical treatment, symptoms, DE location, surgical time, hospital stay and difference in pre and post hemoglobin levels were collected. The Clavien–Dindo classification was used to assess surgical complications, and multivariate analysis was performed to compare patients with and without adenomyosis. 157 DE patients were included into the study; 77 (49.05%) had adenomyosis according to transvaginal ultrasound (TVS) and were classified in the A group, and 80 (50.95%) had no adenomyosis and were classified in the noA group. Adenomyosis was associated with a higher rate of surgical complications: 33.76% (A group) vs. 12.50% (noA group) (p < 0.001). Multivariate analysis showed a 4.56-fold increased risk of presenting complications in women with adenomyosis (CI 1.90–11.30; p = 0.001) independently of undergoing hysterectomy. There was a statistically significant association between the number of criteria of adenomyosis present in each patient and the proportion of patients presenting surgical complications (p < 0.001). Adenomyosis is an independent preoperative risk factor for surgical complications in DE surgery after adjustment for known demographic, clinical and surgical risk factors.

## Introduction

Adenomyosis is a benign uterine condition defined as the presence of endometrial glands and stroma within the myometrium^1^. Deep endometriosis (DE) was defined as endometriosis infiltrating the peritoneum by > 5 mm^2^. For years endometriosis and adenomyosis were related, but they are now mainly considered as separate entities^3,4^. Deep endometriosis (DE) occurs in 15–30% of patients with endometriosis and is associated with concomitant adenomyosis in around 25–50% of cases^5,6^.

Advances in pelvic ultrasound provide a high accuracy in the diagnosis of the different forms of endometriosis^7^ and adenomyosis^1,8,9^ and allow topographical planning prior to surgery^10,11^.

Hormone therapy is the first line treatment for DE and adenomyosis in patients not seeking pregnancy^12,13^. When severe pain persists following medical treatment, or in cases of intestinal or ureteral obstruction, conservative or radical surgical approaches may be necessary depending on the need for fertility preservation^14,15^. Surgery has been widely demonstrated to improve endometriosis-related symptoms^16^. Some previous studies have shown that pelvic pain was significantly associated with concomitant adenomyosis in patients with DE^17^. Moreover, after surgical treatment of DE, pelvic pain and abnormal uterine bleeding (AUB) were significantly more likely to persist with the presence of adenomyosis”^18^.

Resection of endometriotic lesions is often challenging. Most DE interventions are highly complex and are associated with a significant risk of complications that must be preoperatively taken into account^19^. Moreover, there is no a reliable preoperative marker to determine the severity of endometriosis for extrapolation to surgical difficulty^20^. To the best of our knowledge, there are no data about the effect of the presence of adenomyosis in terms of surgical outcomes and complications.

Thus, the aim of the present study was to assess the impact of adenomyosis on the presentation of surgical complications in patients with DE undergoing laparoscopic surgery.

## Materials and methods

A retrospective cohort study including women referred to the Endometriosis Unit of the Hospital Clinic of Barcelona, who underwent DE surgery from July 2018 to December 2019 was designed.

The study was approved by the Ethical Committee of the Hospital Clinic (Reg: HCB/2019/1152), all research was performed in accordance with relevant guidelines and regulations and informed consent was obtained from all patients.

A preoperative diagnosis of DE and adenomyosis was made by two expert sonographers within 6 months prior to surgery. DE was described according to the fourth step method suggested by the International Deep Endometriosis Analysis (IDEA) group^7^ with a 2-dimensional and 3-dimensional transvaginal sonography (TVS) using an endovaginal probe (type RIC5-9, Voluson V730 Expert; GE Healthcare, Milwaukee, WI) with previous bowel preparation^21^. The location and extent of DE was described within the pelvis: rectovaginal septum, torus uterinus, uterosacral ligaments, vaginal fornix, bladder, ureteral and bowel involvement and the mean maximum size for each DE nodule was evaluated^22^. Adenomyosis ultrasound features according to the criteria of the Morphological Uterus Sonographic Assessment (MUSA) group^8^, were: asymmetrical thickening, cysts, hyperechoic islands, fan-shaped shadowing, echogenic subendometrial lines and buds, translesional vascularity, irregular junctional zone and interrupted junctional zone. Adenomyosis was diagnosed when at least 3 of the above-mentioned ultrasound features were present according to our hospital protocol.

DE surgery was always performed by the same team of skilled endometriosis surgeons and a colorectal surgeon or urologist when needed. In order to standardize all the surgical procedures, they were classified into: adnexal (including salpingectomy, ovarian cystectomy or CO2 laser vaporization and adnexectomy), pelvic (vagina, uterosacral ligaments, torus uterinus and rectovaginal septum), bowel (shaving, discoid or segmental resection), urinary (ureterolysis, bladder nodule excision, nephrectomy) and hysterectomy. Some patients underwent more than one of these procedures.

After preoperative assessment, surgery was performed as follows: first, in cases of hysterectomy, a uterine manipulator (Hohl, Karl Storz, Tuttlingen, Germany) was placed. Surgery was begun with the dissection of rectosigmoid adhesions through the pelvic rim with the opening of the retroperitoneum bilaterally in order to control the entire pathway of the ureters. After that, we proceeded to open and dissect bilateral pararectal spaces to individualize DE nodules, avoiding hypogastric plexus injury; the medial pararectal space was dissected between the mesorectum space, the rectal pillars and the uterosacral ligaments and laterally to the mesoureter creating the lateral para rectal fossa. Dissection of the rectovaginal septum (up to recognizable healthy areolar tissue) allowed vaginal and rectal nodule excision. Then, bowel, ureteral, parametrial, uterine (torus, USL) or vaginal nodules were identified and subsequently removed according to the specific surgical technique. In patients undergoing hysterectomy we then performed the transection of the uterovarian or infundibulopelvic ligaments (depending on bilateral adnexectomy or not), the transection of round ligaments, which had previously been cut to better access to pararectal spaces; caudally, both ureteral tunnels were identified. The uterine vessels were dissected and cut at the cross-point of the ureter. The spaces between the rectum and the endometriotic lesions were previously identified and dissected, which allowed skeletonization and transection of the posterior parametrium. Finally, the cardinal ligaments were transected and colpotomy was made through the uterine manipulator.

If the ovaries were affected by ovarian endometriomas they were drained as a first surgical step (to better prepare space or visualization) and at the end of surgery, after removal of the DE nodules, OMAS were treated by stripping or ablation with CO_2_ laser (in order to preserve ovarian reserve).

In addition, endometriosis was staged according to the revised-American Society of Reproductive Medicine (r-ASRM) classification score^23^.

The following demographic data were collected: age, body mass index (BMI), previous endometriosis surgery, infertility, parity, preoperative hormonal treatment and surgical indication. The endometriosis-related symptoms considered were: dysmenorrhea, dyspareunia, dyschezia, dysuria and non-cyclic pelvic pain, using a numerical rating scale (NRS) in which 0 was no pain and 10 unbearable pain. Abnormal uterine bleeding (AUB) was also registered.

Patients were divided into two groups according to the preoperative TVS features: with adenomyosis (A) or without adenomyosis (noA). The main goal of the study was to compare postoperative complications in the two groups according to the Clavien-Dindo (CD) classification^24^. Differences in demographic characteristics, medical treatment, symptoms, DE location, types of DE surgery, surgical time (minutes), hospital stay (days) and pre and post hemoglobin levels (gr/dl) were also assessed.

### Statistical analysis

Statistical analysis was performed using SPSS v 21.0 software (IBM, Armonk, NY, USA). Patient characteristics were described using frequency tables for nominal variables and measures of central tendency and dispersion for continuous variables.

To compare outcomes between the two groups, the Chi-squared or Fisher exact tests was used for categorical data, and continuous variables were compared using independent t-tests or the Mann–Whitney test as appropriate. Bivariate logistic regression analysis was used to determine the factors related to the incidence of surgical complications. All bivariate statistical tests were performed at a significance level of p < 0.05 (two-sided). In order to identify a logistic predictive regression model, clinical and statistical judgment led to the assessment of the following independent variables that were related to complications in the bivariate analysis with a p < 0.15: adenomyosis, surgical time, bowel resection and hysterectomy.

This model explained the probability of the presentation of surgical complications with respect to non-presentation of complications as a function of the variables included. For the multivariate analysis, up to p < 0.10 was illustrated.

Furthermore, we also conducted a subanalysis of the complications between groups considering patients without hysterectomy.

## Results

A total of 157 patients undergoing DE surgery were included during the study period. According to TVS, 77 (49.05%) patients had 3 or more adenomyosis criteria and were classified in the A group and 80 (50.95%) patients had less than 3 adenomyosis criteria and were classified in the noA group.

No differences were observed between groups regarding age, BMI and parity (Table 1). A total of 52.86% of women had one or more previous endometriosis surgeries, with no significant differences between groups. Among the 157 patients studied, 130 (82.81%) received preoperative continuous hormonal medical treatment, mainly with oral combined contraceptives, with no significant differences between groups.

**Table 1:** Clinical characteristics and endometriosis-related symptoms.

	Adenomyosis (A) N = 77	No adenomyosis (noA) N = 80	p value
Age, years (mean ± SD)	38.22 ± 6.63	37.64 ± 5.85	NS
BMI kg/m^2^ (mean ± SD)	24.45 ± 4.98	23.90 ± 5.41	NS
Parity (mean ± SD)	0.43 ± 0.81	0.41 ± 0.83	NS
Infertility**	36 (46.75)	28 (35)	NS
**Hormonal treatment n (%)**			
None	14 (18.18)	13 (16.25)	NS
Combined contraceptives	32 (41.55)	36 (45)	
Progestins	13 (16.88)	13 (16.25)	
LNG-IUD	5 (6.49)	3 (3.75)	
aGnRH	13 (16.88)	15 (18.75)	
**DE location n (%)**			
Torus uterinus	49 (63)	27 (33.75)	NS
Vaginal fornix	16 (20.77)	10 (12.50)	
Uterosacral ligaments	44 (57.14)	32 (40)	
Rectovaginal septum	5 (6.49)	6 (7.50)	
Ureteral + bladder	14 (18.18)	12 (15)	
Bowel	35 (45.45)	32 (40)	
Ovarian endometrioma n(%)	60 (77.9)	52 (65)	
**Maximum size of DE nodules (mean ± SD)**			
Torus uterinus	14.35 ± 6.09	13.77 ± 5.87	NS
Vaginal fornix	11.24 ± 5.68	12.05 ± 6.12	
Uterosacral ligaments	12.76 ± 4.77	11.55 ± 4.32	
Rectovaginal septum	21.14 ± 7.90	19.23 ± 6.78	
Ureteral + bladder	22.32 ± 6.41	23.12 ± 7.30	
Bowel	33.81 ± 9.72	34.92 ± 8.98	
**Uterine corpus size (mean ± SD)**			
Length	69.91 ± 8.42	69.54 ± 7.20	NS
Anteroposterior	35.11 ± 5.45	34.33 ± 6.12	
Transverse	46.20 ± 12.31	43.92 ± 9.82	
**Previous endometriosis surgery n (%)**			
None	33 (42.85)	41 (51.25)	NS
1 procedure	34 (44.15)	33 (41.25)	
≥ 2 procedures	10 (12.98)	6 (7.50)	
**AUB n (%)**			
No	48 (62.33)	51 (63.75)	NS
Yes	29 (37.66)	29 (36.25)	
Dysmenorrhea* (mean ± SD)	7.20 ± 3.13	6.85 ± 3.03	NS
Dyspareunia (mean ± SD)	4.72 ± 4.05	4.61 ± 3.84	NS
Dyschezia (mean ± SD)	3.60 ± 3.87	3.88 ± 3.88	NS
Non-cyclic pelvic pain (mean ± SD)	4.69 ± 3.50	4.09 ± 3.49	NS
Dysuria (mean ± SD)	1.22 ± 2.81	1.02 ± 2.41	NS

Data are given as n or %. Symptoms are expressed with Numeric Rating Scale (NRS). *LNG-IUD* Levonorgestrel Intra Uterine Device, *aGnRH* analog-Gonadotropin-releasing Hormone, *SD* standard deviation, *n* number, *%* percentage, *AUB* Abnormal Uterine Bleeding, *NS* non-significant, *BMI* body mass index.

*Dysmenorrhea NRS was reported in patients with regular monthly periods: 14 patients in the A group (18.18%) and 13 in the noA Group (16.25%).

**Infertility: was defined as the failure to achieve pregnancy after 12 months without investigations or treatment.

Table 1 shows the endometriosis-related symptoms of both study groups**.** The mean of all NRS scores and the presence of AUB, although not significant, were higher (except for dyschezia) in the group of patients with adenomyosis (group A). The main indication for surgery was pain in both groups (96%) despite receiving medical treatment, with 46.75% (group A) and 35% (group no A) having associated infertility (p 0.495). The main indications for patients undergoing hysterectomy without adenomyosis were: uterine fibroids (9), persistence of abnormal uterine bleeding despite hormonal treatment (6) and patient's preference not to preserve the uterus (3).

The different interventions performed in the two groups are shown in Table 2. All the procedures were performed by laparoscopy with 56 hysterectomies being registered: 38 in the A group and 18 in the noA group (p 0.002). We found parametrial involvement in 5.09% (8/157) of patients (5 A i 3 noA), with no differences between groups and almost 17% of patients were diagnosed with ureteral DE during surgery.

**Table 2:** Surgical procedures and r-ASRM classification.

	Adenomyosis (A) N = 77	No adenomyosis (noA) N = 80	p value
**Type of surgery n (%)**			
Adnexal surgery*	72 (93.5)	67 (83.75)	NS
Pelvic DE^+^	55 (71.42)	45 (56.25)	NS
Bowel Surgery			
Shaving	22 (28.57)	19 (23.75)	NS
Segmental resection	13 (16.88)	13 (16.25)	NS
Urinary surgery			
Bladder nodule excision	5 (6.49)	6 (7.5)	NS
Ureterolysis/reimplantation	9 (11.68)	6 (7.5)	NS
Hysterectomy: 56/157 (35.66)	38 (49.35)	18 (22.5)	**0.002**
**r-ASRM score n (%)**			
I	3 (3.89)	1 (1.25)	NS
II	4 (5.19)	9 (11.25)	
III	12 (15.58)	19 (23.75)	
IV	58 (75.32)	51 (63.75)	

Significant values are in bold.

*Adnexal surgery: unilateral or bilateral including ovarian endometriomas, salpingectomy, adnexectomy. ^+^Pelvic DE: including vagina, uterosacral ligaments, torus uterinus.

Data are given as n or %. *r-ASRM score* revised-American Society of Reproductive Medicine, *SD* standard deviation, *n* number, *%* percentage, *NS* non-significant.

DE was confirmed histologically in all cases as well as the presence of adenomyosis when hysterectomy or uterus-sparing adenomyosis surgery was performed. In 2 out of 18 cases (11.11%) we found incidental small foci of adenomyosis in the histopathologic post-operative analysis of patients in the no A group.

The presence of adenomyosis showed significant differences in the CD complications rates: A group 33.76%, noA group 12.50% (p = 0.001), mean surgical time: A group 231 ± 101 min, noA group 181.08 ± 91.61 min (p = 0.011) and difference in pre-post hemoglobin levels: A group 2.17 ± 1.89 g/dl, and noA group 2.05 ± 1.22 g/dl (p = 0.049). There were no significant differences in length of hospital stay: A group 3.32 ± 3.70 days, noA group 2.75 ± 1.85 days (p = 0.09). Most of the complications were minor (88.8% CD I and II). Sixteen patients presented CD II complications including: anemia requiring blood transfusion (5), urinary infection (3), hematoma of the vaginal vault (4), wound trocar infection (2) and intestinal subocclusion (2) (Table 3). Four patients in the A group, presented major complications (CD III): one rectovaginal fistula, two anastomotic leakages and one bowel obstruction. All patients were recovered at 3–6 months of post-operative follow-up.

**Table 3:** Surgical data and complications.

	Adenomyosis (A) N = 77	No adenomyosis (noA) N = 80	p value
Surgical time minutes (mean ± SD)	231 ± 101	181.08 ± 91.61	**0.011**
Difference pre-post Hemoglobin levels g/dl (mean ± SD)	2.17 ± 1.89	2.05 ± 1.22	**0.049**
Hospital stay days (mean ± SD)	3.32 ± 3.70	2.75 ± 1.85	0.09
**CD complications**			
n (%)	26 (33.76)	10 (12.5)	**0.001**
I	12 (15.58)	4 (5)	
II	10 (12.98)	6 (7.5)	
III	4 (5.19)	0 (0)	
IV	0 (0)	0 (0)	

Significant values are in bold.

Data are given as n or %. *SD* standard deviation, *CD* Clavien–Dindo, *n* number, *%* percentage.

**Figure 1:**
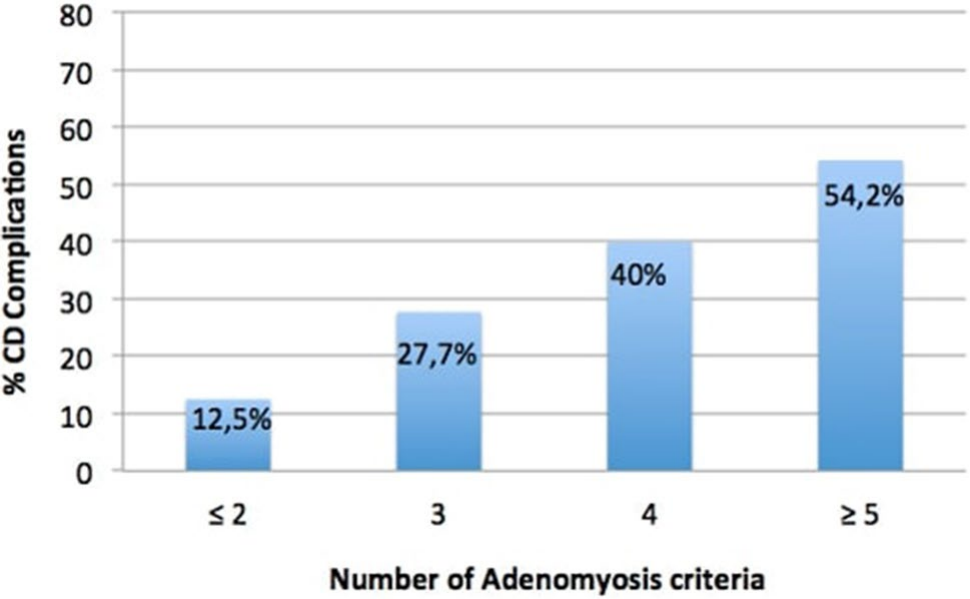
Association between adenomyosis criteria and surgical complications.

Multiple logistic regression showed the following factors to be independently related to the development of surgical complications: adenomyosis, surgical time, bowel resection and hysterectomy. The attributed risk of each factor is shown in Table 4. The risk of presenting complications increased 4.56 times in the A group (CI 1.90–11.30; p = 0.001). Moreover, there was a statistically significant association between the number of criteria of adenomyosis and the proportion of patients presenting a surgical complication (p < 0.001) (Fig. 1).

**Table 4:** Risk factors related to surgical complications: multivariate logistic regression analysis.

	Odds ratio	95% CI	p value
Adenomyosis	4.558	1.845–11.26	0.001
Surgical time	1.010	1.004–1.016	0.002
Bowel resection	2.558	0.843–7.761	0.097
Hysterectomy	3.110	1.293–7.478	0.011

*CI* confidential interval.

A higher CD complication rate was also observed in the A group when excluding patients with hysterectomy in both groups (Table 5).

**Table 5:** Surgical data considering patients without hysterectomy.

	Adenomyosis (A) N = 39	No adenomyosis (noA) N = 62	p value
Surgical time minutes (mean ± SD)	226.1 ± 105.7	185.52 ± 81.35	**0.032**
Difference pre-post Hemoglobin levels g/dl (mean ± SD)	2.22 ± 1.41	1.85 ± 1.11	0.175
Hospital stay days (mean ± SD)	3.85 ± 4.59	2.58 ± 1.76	**0.02**
**CD complications**			
n (%)	13 (33.33)	5 (8)	**0.003**
I	7 (17.94)	3 (4.83)	
II	5 (12.82)	2 (3.22)	
III	1 (2.56)	0 (0)	
IV	0 (0)	0 (0)	

Significant values are in bold.

Data are given as n or %. *SD* standard deviation, *CD* Clavien–Dindo, *n* number, *%* percentage.

## Discussion

The possibility of identifying predictable preoperative features that could modify surgical results would be of great interest^25^. Previous studies^26,27^ have already described a higher rate of complications depending on DE severity, although in our patients, the nodule size was not related. The results of our study suggest, for the first time, that the presence of adenomyosis may contribute to increasing the surgical complication rate in DE patients. Furthermore, this increased risk is related to the number of TVS adenomyosis criteria.

While the impact of adenomyosis on surgical results has been assessed in other benign conditions, to our knowledge no previous study has evaluated the impact of adenomyosis on surgical complications in women with DE undergoing laparoscopic surgery^28^. Previous studies in benign pathology reported an increased rate of bladder and ureteral complications for vaginal hysterectomy in patients with only adenomyosis^29^ but this was not observed with the laparoscopic approach^30^. Accurate preoperative imaging assessment has been described in cases of bowel DE, and several previous studies have reported a high accuracy of up to 89.90% and 98.10% in correlating TVS findings with surgical difficulties^11,20^. These studies are primarily based on ultrasound findings of ovarian mobility and the presence or not of bowel endometriosis but did not specifically included the presence of adenomyosis.

It has been shown that several 2D and 3D TVUS features are associated with adenomyosis; however the importance of each item, the minimum number of items or their combination in the diagnosis of adenomyosis has yet to be defined^1,31^. Indeed, there is no current consensus on the criteria for the diagnosis of adenomyosis by TVUS. In order to improve accuracy, in our study we considered adenomyosis when 3 of the MUSA criteria were present. Some authors^32^ also evaluated the diagnostic accuracy of the question mark sign and TVUS uterine tenderness in the diagnosis of adenomyosis, concluding that they are also useful tools.

Due to the surgical complexity of DE procedures, even in expert hands, the rate of complications is still considerable especially when colorectal resection is involved, ranging from 3.40 to 25%^25,33,34^. The use of endovenous ICG during surgery for rectosigmoid or ureteral endometriosis has recently been proposed to assess blood perfusion of the bowel during anastomosis and the ureter blood supply, in order to guide surgical decisions and avoid complications^35,36^. In our study, clinically relevant complications (CD type III) were only recorded in 2.54% of cases. These complications were also related to bowel surgeries, similar to previously published data^15,33,34,37^.

Hysterectomy is considered the surgical treatment of choice for most women with adenomyosis who do not wish to preserve fertility after medical treatment has failed^38^. Concomitant hysterectomy in the A group was performed in 49.35% vs. 22.50% in the noA group (p = 0.002). An explanation for this high proportion of hysterectomy may be that our hospital is a referral center which receives complex patients, with a high percentage of previous pelvic surgeries (52.86%), and thus, a high number of women are elective for a definitive surgery undergo elective surgery. In the context of DE hysterectomy has been shown to be associated with a longer operative time^39^ and per se may be an important confounder for the presence of postoperative complications. However, the application of a multivariate model demonstrated that hysterectomy was an independent factor with or without adenomyosis. This was also confirmed in the subanalysis of surgical outcomes between groups performed considering only patients without hysterectomy. Interestingly, the history of previous surgery was not related to a higher risk of complications likely due to our small sample size.

Arena et al.^40^ reported that factors such as previous endometriosis surgery, the presence of adenomyosis and parametrial location may increase the risk of intraoperative or postoperative complications in cases of ureteral endometriosis surgery. In our study all the factors that could contribute to a higher risk of complications (bowel and urinary surgery, previous endometriosis surgeries and operative time) were adjusted in the multivariate analysis in order to prevent bias.

On the other hand, Van den Bosch et al.^9^ proposed a new classification system which includes different adenomyosis subtypes, including intrinsic/extrinsic adenomyosis, adenomyosis external and focal adenomyosis in the outer myometrium (FAOM)^5,41^. Focal adenomyosis commonly affects the external part of the myometrium and may attach the uterus to other structures, hindering surgery and increasing the risk of presenting surgical complications and impairing other surgical outcomes and may be a possible explanation for our study results although a definite reason is unknown. Moreover, the chronic inflammation, proliferation and fibrosis that supports the pathogenesis of adenomyosis^1^ may be another feasible explanation, creating an inflammatory environment more likely to present an increased risk of surgical complications. Further prospective studies are needed to support this correlation with the type of adenomyosis (since this classification was published after performing the present study) and to find a pathogenic mechanism explaining the clinical presentation of our surgical results.

This study has several strengths: it is the first to describe the impact of adenomyosis on surgical outcomes by conditioning the presentation of a surgical complication. Second, the TVS evaluation was performed by two expert sonographers who have previously demonstrated a high diagnostic TVS accuracy for determining the presence of DE (sensitivity 100%, specificity 96%)^21^. Moreover, all surgeries were performed by the same team of experienced surgeons in advanced laparoscopy surgery focused on endometriosis.

However, some limitations need to be acknowledged. This was a retrospective, single-center study. In the absence of previous studies, no formal power calculation was performed for sample size determination. The limitation of our small sample size can probably explain why previous surgeries and bowel surgery were not determinant factors in the rate of complications in our study. Secondly, a patient selection bias may be present. The patients were probably more complex since they were referred to the Endometriosis Unit of a tertiary university center and were, therefore, associated with a higher degree of surgical difficulty and may not be comparable with the general population. Another limitation of our study, is that patients were staged according to the r-ASRM classification score that correlates poorly with pain and surgical complexity. Recently, an anatomy-based and user-friendly scoring system that correlates with surgical complexity has been developed by the American Association of Gynecologic Laparoscopists^42^, and its use should be considered in future studies to predict surgical difficulty and avoid complications.

## Conclusion

According to our findings, adenomyosis is an independent preoperative risk factor for surgical complications after adjustment for known demographic, clinical and surgical risk factors. Thus, patients with a preoperative diagnosis of DE and adenomyosis programmed to undergo surgery should be informed about the possible development of surgical complications and ideally attended in a referral center by an expert multidisciplinary endometriosis team. However, further well-designed prospective studies should be carried out in order to confirm these findings taking into account different adenomyosis phenotypes and DE severity, and including patients without hysterectomy.
